# BcMettl4-Mediated DNA Adenine N^6^-Methylation Is Critical for Virulence of *Botrytis cinerea*

**DOI:** 10.3389/fmicb.2022.925868

**Published:** 2022-06-30

**Authors:** Zhengang Miao, Guangyuan Wang, Heng Shen, Xue Wang, Dean W. Gabriel, Wenxing Liang

**Affiliations:** ^1^College of Plant Health and Medicine, Engineering Research Center for Precision Pest Management for Fruits and Vegetables of Qingdao, Shandong Engineering Research Center for Environment-Friendly Agricultural Pest Management, Shandong Province Key Laboratory of Applied Mycology, Qingdao Agricultural University, Qingdao, China; ^2^College of Life Sciences, Shandong Province Key Laboratory of Applied Mycology, Qingdao Agricultural University, Qingdao, China; ^3^Yantai Agricultural Technology Extension Center, Yantai, China; ^4^Department of Plant Pathology, University of Florida, Gainesville, FL, United States

**Keywords:** *Botrytis cinerea*, 6mA, methyltransferase, MeDIP-Seq, virulence

## Abstract

DNA adenine N^6^-methylation (6mA) plays a critical role in various biological functions, but its occurrence and functions in filamentous plant pathogens are largely unexplored. *Botrytis cinerea* is an important pathogenic fungus worldwide. A systematic analysis of 6mA in *B. cinerea* was performed in this study, revealing that 6mA is widely distributed in the genome of this fungus. The 2 kb regions flanking many genes, particularly the upstream promoter regions, were susceptible to methylation. The role of BcMettl4, a 6mA methyltransferase, in the virulence of *B. cinerea* was investigated. *BcMETTL4* disruption and point mutations of its catalytic motif “DPPW” both resulted in significant 6mA reduction in the genomic DNA and in reduced virulence of *B. cinerea*. RNA-Seq analysis revealed a total of 13 downregulated genes in the disruption mutant ΔBcMettl4 in which methylation occurred at the promoter sites. These were involved in oxidoreduction, secretory pathways, autophagy and carbohydrate metabolism. Two of these genes, *BcFDH* and *BcMFS2*, were independently disrupted. Knockout of *BcFDH* led to reduced sclerotium formation, while disruption of *BcMFS2* resulted in dramatically decreased conidium formation and pathogenicity. These observations indicated that 6mA provides potential epigenetic markers in *B. cinerea* and that BcMettl4 regulates virulence in this important plant pathogen.

## Introduction

DNA methylation is one of the basic epigenetic markers involved in regulation of various biological functions in both prokaryotes and eukaryotes (Bird, 2007; Vasu and Nagaraja, 2013). Among the methylation modifications, DNA adenine N^6^-methylation (6mA) and 5-methylcytosine (5mC) are two of the most important. The 5mC modification has been well studied as an epigenomic marker in eukaryotes (Zemach et al., 2010), while adenine N^6^-methylation has been found to be predominantly distributed in prokaryotic genomes (Blow et al., 2016) and eukaryotic RNA (Fu et al., 2014). In recent years, 6mA has also been found to be widespread in the genomic DNA of many eukaryotes, including fungi (Mondo et al., 2017), *Chlamydomonas* (Fu et al., 2015), *Caenorhabditis elegans* (Greer et al., 2015), *Arabidopsis thaliana* (Liang et al., 2018), rice (Zhou et al., 2018) and humans (Xiao et al., 2018). However, compared with those in prokaryotes, the characteristics of 6mA methylation in eukaryotes are still largely unknown.

Previous studies have shown that 6mA is dynamically controlled by methyltransferase and demethylase (Greer et al., 2015; Zhang et al., 2015). In prokaryotes, M.*Mun*I is a classic 6mA methyltransferase (Iyer et al., 2016). It has been well documented that the orthologs of MT-A70 that evolved from M.*Mun*I (Iyer et al., 2011) in eukaryotes are important 6mA methyltransferases (Bujnicki et al., 2002). Three MT-A70 methyltransferases, namely, Mettl4 in *Bombyx mori* (Wang et al., 2018) and Mettl3 and Mettl14 in humans (Wang et al., 2016), have been shown to play important roles in regulating the levels of 6mA. Mta1, an MT-A70 ortholog in *Oxytricha*, can form a dimer with Mta9 and has the function of a DNA methyltransferase (Woodcock et al., 2020). We found an MT-A70 homologous protein, BcMettl4, in *Botrytis cinerea*, but the function of this protein in DNA methylation was unclear.

*Botrytis cinerea* is an important necrotrophic fungal pathogen that infects over 1,000 plants and causes gray mold disease. Many economically important vegetables and fruits, including strawberry, tomato, cucumber and grape, are susceptible to *B. cinerea*. *B. cinerea* is ranked as the second most common plant pathogenic fungus in the world (Dean et al., 2012) and causes severe preharvest and postharvest economic losses worldwide (Mousavi-Derazmahalleh et al., 2019). However, the biological role of the 6mA DNA modification in *B. cinerea* is still unclear.

In this study, the 6mA DNA modification in *B. cinerea* was investigated and 6mA was found to be widely distributed in *B. cinerea*. The gene *BcMETTL4* was disrupted in the wild-type strain B05.10. Methylated DNA immunoprecipitation sequencing (MeDIP-Seq) was further employed to analyze the methylome characteristics of the wild-type strain B05.10 and the gene disruption mutant ΔBcMettl4. Deletion of *BcMETTL4* caused a global reduction in DNA 6mA. RNA-Seq analysis was subsequently used to investigate global gene expression patterns in the disruption mutant ΔBcMettl4 and its wild-type strain B05.10. Finally, the relationships between 6mA, gene expression and virulence were investigated. Expression of key genes involved in virulence and conidiation was strongly affected by 6mA modification in *B. cinerea*.

## Materials and Methods

### Strains and Growth Assays

Strain B05.10 of *B. cinerea* used in this study was isolated from *Vitis vinifera* (Yang et al., 2018). The wild-type strain, gene deletion derivatives and complemented strains were grown on PDA plates (20% potato, 2% dextrose, and 1.5% agar). The growth ratios, conidial development, and sclerotium formation of different strains of *B. cinerea* were measured according to previously described methods (Yang et al., 2019). For mycelial growth assays, the different strains were cultured at 25°C for 3 day on PDA plates supplemented with 0.5 mM CR, 0.73 mM SDS, 20 mM H_2_O_2_, 1 M sorbitol, 1 M KCl, 1 M NaCl, and 1 M glycerine.

### Construction of Gene Deletions, Complementation, Subcellular Localization and Point Mutations

To construct the gene mutant cassette for targeted deletions, two flanking sequences of each gene were obtained from genomic DNA of strain B05.10 by PCR. The hygromycin B phosphotransferase (*HPH*) gene was derived from the plasmid pBS-HPH1 (Yang et al., 2018). The two flanking fragments and the *HPH* gene were fused by splice overlap PCR. The resulting deletion cassettes (*Promoter:HPH:Terminator*) were connected to the pMD19-T vector for sequencing. The gene deletion cassette was transformed into strain B05.10.

For complementation, full-length genes, including promoters and terminators, were amplified from the B05.10 genome. The resulting PCR products were cloned into plasmid pBS-neo (Yang et al., 2018). The resulting vectors were subsequently transformed into the deletion mutants.

To construct BcMettl4-GFP, BcDamt-GFP, and BcMettl4^Δ^ *^NLS^*-GFP fusion cassettes, the open reading frames (ORFs) of *BcMETTL4* and *BcDAMT* without the stop codons were amplified by PCR from strain B05.10. The NLS of BcMettl4 was deleted using overlap PCR (Heckman and Pease, 2007). The PCR products were ligated into plasmid pNAH-OGG-G418 that was derived by replacing the hygromycin B resistance gene of pNAH-OGG (Schumacher, 2012) with a geneticin G418 resistance gene from plasmid pBS-neo (Yang et al., 2018).

For generation of the BcMettl4 point mutant, we first designed primers that contained the mutated site. The sites upstream and downstream of the mutated site were obtained by PCR and the products were joined by overlap PCR. The resulting PCR products were cloned into the plasmid pBS-neo and transformed into the ΔBcMettl4 strain using standard protoplast formation procedures as previously described (Gronover et al., 2001; Yang et al., 2018).

All primers and their descriptions are shown in Supplementary Table 1. Positive transformants were selected on PDA plates with 0.01% hygromycin B or 0.01% geneticin G418 sulfate. PCR was used to confirm the positive transformants.

### Dot Blot Assay

Genomic DNA from various strains was extracted using a Fungi Genomic DNA Purification Kit (Sangon Biotech, China). After denaturation at 98°C for 5 min, the extracted DNA was chilled on ice for 10 min and then transferred to a HybondTM-N + membrane. After drying at room temperature, the DNA was fixed to the membrane by UV cross-linking. The HybondTM-N + membrane was blocked in 5% skim milk powder for 1.5 h at room temperature, followed by incubation with 6mA antibody (1:2,000 dilution, Abcam, ab151230) for 10 h at 4°C. The membrane was then washed four times for 8 min using TBST [25 mM Tris–HCl (pH 7.4), 140 mM NaCl and 0.1% Tween-20]. After incubation with secondary antibody (1:5,000 dilution, Sigma A0545) at room temperature for 2 h, the membrane was washed 4 times for 8 min using TBST. Finally, an ECL Immunoblotting Detection Kit (Thermo Fisher) was used for signal visualization. The input DNA was normalized by using methylene blue.

### MeDIP-Seq

MeDIP-Seq was performed according to previously described protocols (Fu et al., 2015; Chen et al., 2018; Zhou et al., 2018). In brief, the genomic DNAs of different strains were extracted from conidia grown in YEPD medium for 24 h at 25°C, followed by incubation with RNase A overnight. After sonication, the DNA fragments (200–400 bp) were repaired, followed by adaptor ligation. The resulting DNA was purified using a TIANquick Midi Purification Kit (DP204, TIANGEN BIOTECH, China). The purified DNA fragments were denatured at 94°C for 15 min and chilled on ice for 10 min. An aliquot of denatured DNA was used as input. The remaining DNA was incubated with anti-6mA antibody at 4°C for 12 h. After elution by 6mA salt competition, a 6mA-IP-Seq library was constructed by PCR amplification. The resulting DNA fragments were sequenced with a HiSeq 2500 (Illumina) (Zhou et al., 2018). Raw reads were trimmed to remove adaptors and low-quality bases, and the reads with 6mA-rich regions were called with MASC2 (Liu, 2014).

### RNA-Seq Analysis

After conidial germination in YEPD medium at 25°C for 24 h, the mycelia of different strains were collected by centrifugation for RNA extraction. Total RNA was extracted using TRIzol Reagent (Invitrogen). A NanoDrop spectrophotometer (Thermo Scientific) was employed to determine the RNA concentration, quality and integrity. Poly-T oligo-attached magnetic beads were purified to enrich mRNA. First-strand cDNA was obtained using random oligonucleotides and SuperScript II. Second-strand cDNA synthesis was subsequently performed using DNA polymerase I and RNase H. The resulting cDNA fragments were further adenylated at the 3’ ends and ligated with Illumina PE adaptor oligonucleotides. PCR was performed to enrich the DNA fragments with ligated adaptors on both ends. After purification using an AMPure XP system and quantification using an Agilent high-sensitivity DNA assay, the obtained sequencing library was sequenced on a NovaSeq 6000 platform (Illumina).

### Bioinformatics Analysis

TopHat (v2.0.12) was employed to map the obtained sequences against the database of *B. cinerea* (Trapnell et al., 2009). The mapped genes were calculated using HTSeq (v0.6.1) (Anders et al., 2015). Gene expression was quantitatively estimated by using the reads per kilobase per million mapped reads (RPKM) value (Trapnell et al., 2010). DESeq was used to analyze the differentially expressed genes between the wild-type and the gene deletion mutants (Wang et al., 2010).

### Pathogenicity Experiments on Tomato Leaves and Onion Epidermis

After growth on PDA plates at 25°C for 7 days, the conidia produced by different strains of *B. cinerea* were harvested and resuspended in buffer (6.7 mm K_2_HPO_4_ and 10 mm glucose). A total of 5 μl of conidia suspension (5.0 × 10^5^ conidia/ml) was transferred to 8-week-old tomato (*Solanum lycopersicum*) leaves, incubated at 25°C for 72 h, and developing disease lesions caused by different strains were measured.

Additionally, infection-related morphogenesis was observed on onion epidermis. The conidial suspensions (3.0 × 10^3^ conidia/ml) were inoculated onto the epidermis. After culturing for 12 h at 25°C in the dark, the onion skin was stained with aniline blue and observed.

### Induced Expression of *BcMETTL4* and *BcDAMT* in Interactions Between *Botrytis cinerea* and Tomato Leaves

The expression levels of *BcMETTL4* and *BcDAMT* in wild-type B05.10 in interaction between *B. cinerea* and tomato leaves were investigated by real-time PCR. In brief, 45 ml of conidia suspension (5.0 × 10^6^ conidia/ml) and 5 ml of YEPD medium were mixed and 3 g of sterile fresh intact tomato leaves (3 weeks old) was added, and then gently agitated in a shaker at 25°C. Samples were collected at 6 h intervals for 24 h. RNA was extracted according to a previously described protocol (Yang et al., 2018). cDNA was biosynthesized by reverse transcription using a PrimeScript RT Kit (Takara, Japan). The primers used are shown in Supplementary Table 1. Relative expression levels were calculated using the 2^–ΔΔ*Ct*^ method (Livak and Schmittgen, 2001).

### Western Blot

The mycelia grown in YEPD medium at 25°C for 24 h were collected. A nuclear protein extraction kit (R0050, Solarbio) was used to separate nuclear and cytoplasmic proteins. The proteins were separated by SDS-PAGE and immunoblotted using anti-GFP antibody (ab290, Abcam), anti-H3 antibody (ab1791, Abcam), and anti-TUBA4A antibody (D110022, BBI Life Sciences).

## Results

### Analysis of the Methyltransferases in *Botrytis cinerea*

**FIGURE 1:**
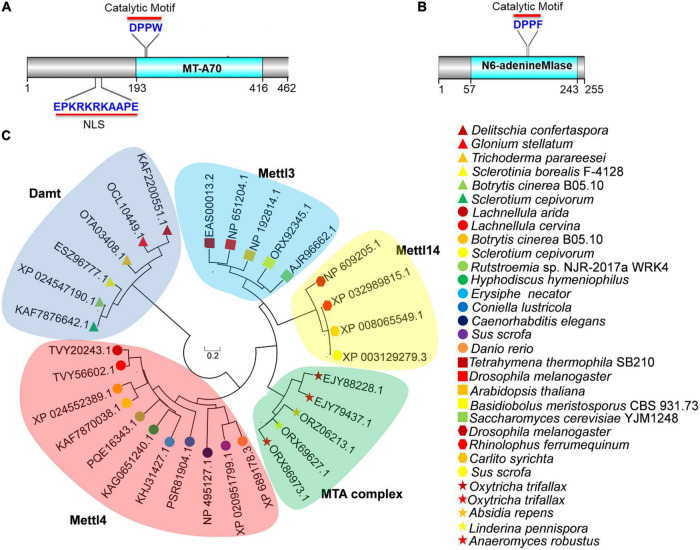
Bioinformatics analysis of BcMettl4 and BcDamt. **(A)** The structural domains encoded by BCIN_12g06440. **(B)** The structural domain encoded by BCIN_02g05970. **(C)** Phylogenetic tree of methyltransferase orthologs based on a neighbor-joining analysis.

To investigate the presence of 6mA in *B. cinerea*, we searched the genome of *B. cinerea* B05.10 using BLAST and found two potential candidates for 6mA methyltransferase. One candidate was BCIN_12g06440 (also known as BC1G_13837), encoding a hypothetical protein (NCBI id: XP_024552389.1) which is a 462-amino-acid ortholog of MT-A70 in *B. cinerea* (Figure 1A). The other was BCIN_02g05970 (also known as BC1G_16157), encoding a hypothetical protein (255 amino acids) containing a N6-adenineMlase (probable N6-adenine methyltransferase) catalytic domain (NCBI id: XP_024547190.1) (Figure 1B). As shown in Figure 1A, the protein encoded by BCIN_12g06440 contained a conserved catalytic motif (“DPPW”) and a nuclear localization signal (NLS). However, the protein encoded by BCIN_02g05970 contained only one catalytic motif, “DPPF” (Figure 1B).

A variety of methyltransferases, including Mta1, Mta9, Mettl3, Mettl4, Mettl14, and Damt, have previously been reported (Wang et al., 2016, 2018; Chen et al., 2018; Beh et al., 2019; Woodcock et al., 2020). A phylogenetic tree of methyltransferase orthologs was constructed using MEGA 7.0 software, revealed that the protein encoded by BCIN_12g06440 in *B. cinerea* was closely related to a branch representing the MT-A70 ortholog Mettl4, while the protein encoded by BCIN_02g05970 belonged to a clade of Damt (Figure 1C). The hypothetical proteins encoded by BCIN_12g06440 and BCIN_02g05970 were thus named BcMettl4 and BcDamt, respectively. The roles of BcMettl4 and BcDamt in the virulence of *B. cinerea* were subsequently studied in some detail.

### Effects of *BcMETTL4* and *BcDAMT* Disruption on Genome Methylation in *Botrytis cinerea*

**FIGURE 2:**
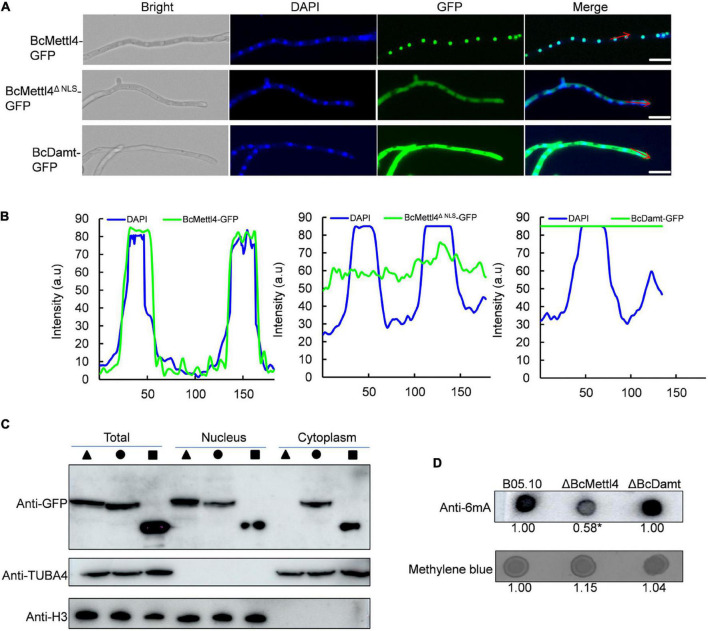
Subcellular localization of BcMettl4 and BcDamt, and effects of *BcMETTL4* and *BcDAMT* deletion on genome methylation. **(A)** Mycelia images for subcellular localization of the BcMettl4-GFP, BcMettl4^Δ^ *^NLS^*-GFP, and BcDamt-GFP. Scale bar = 20 μm. **(B)** Fluorescence intensity of BcMettl4-GFP, BcMettl4^Δ^ *^NLS^*-GFP, and BcDamt-GFP. The blue line represents DAPI while the green line shows GFP. The signal areas were marked by red lines in Figure 2A and the signal quantification were calculated by ImageJ. **(C)** Western blot analysis of BcMettl4-GFP (triangle), BcMettl4^Δ^ *^NLS^*-GFP (circle), and BcDamt-GFP (square). Histone H3 served as a reference for proteins in the nucleus. Alpha tubulin 4a was used as a control for cytoplasmic proteins. **(D)** Dot blot assay for verification the presence of 6mA in the genome DNA of *B. cinerea*. A total of 200 ng DNA was investigated by dot blot assay. Relative 6mA abundance in different strains was calculated by ImageJ. The relative ratio after normalization is to reflect the difference among various treatments. The asterisk means significant difference in values at *p* < 0.05.

Since BcMettl4 contains a predicted NLS signal (Figure 1A), this protein might localize in the nucleus. To test this hypothesis, two fusion proteins, BcMettl4-GFP and BcMettl4^Δ^ *^NLS^*-GFP (without a NLS), were expressed in strain B05.10 of *B. cinerea*. After growth on PDA plates for 3 days, the transformants containing the fusion protein BcMettl4-GFP or BcMettl4^Δ^ *^NLS^*-GFP were observed by fluorescence microscopy. The results showed that the GFP signal of BcMettl4-GFP overlapped with nuclei stained with DAPI fluorescent dye, while BcMettl4^Δ^ *^NLS^*-GFP was present in both the cytoplasm and the nucleus (Figures 2A,B). A western blot analysis was performed using extracted total proteins, nuclear proteins and cytoplasmic proteins and revealed that BcMettl4-GFP was significantly enriched in the nucleus compared with BcMettl4^Δ^ *^NLS^*-GFP (Figure 2C). These findings confirmed that BcMettl4 was located in the nucleus and that the NLS of BcMettl4 played an important role in controlling the subcellular localization of BcMettl4. By contrast, the fusion protein BcDamt-GFP was mainly located in the cytoplasm (Figures 2A–C).

The genes *BcMETTL4* and *BcDAMT* were disrupted in the wild-type strain B05.10. PCR amplification revealed that one transformant, ΔBcMettl4, exhibited *BcMETTL4* gene disruption (Supplementary Figure 1), and another transformant, ΔBcDamt, exhibited *BcDAMT* gene deletion (Supplementary Figure 2). To investigate the presence of 6mA DNA modification in *B. cinerea*, the genomic DNAs (gDNAs) of B05.10, ΔBcMettl4, and ΔBcDamt were extracted and a dot blot assay performed using a specific 6mA antibody. As shown in Figure 2D, a strong immune blot signal was detected in the gDNA of B05.10. Compared with those of B05.10 and ΔBcDamt, the signal in the gene deletion mutant ΔBcMettl4 was significantly weaker, based on ImageJ analysis (*p* < 0.05) (Figure 2D).

Taken together, these observations suggested that 6mA was a widespread DNA modification in the genome of *B. cinerea* and that BcMettl4 may play a role in modifying nuclear DNA.

### Impacts of *BcMETTL4* and *BcDAMT* Disruption on Virulence of *Botrytis cinerea*

**FIGURE 3:**
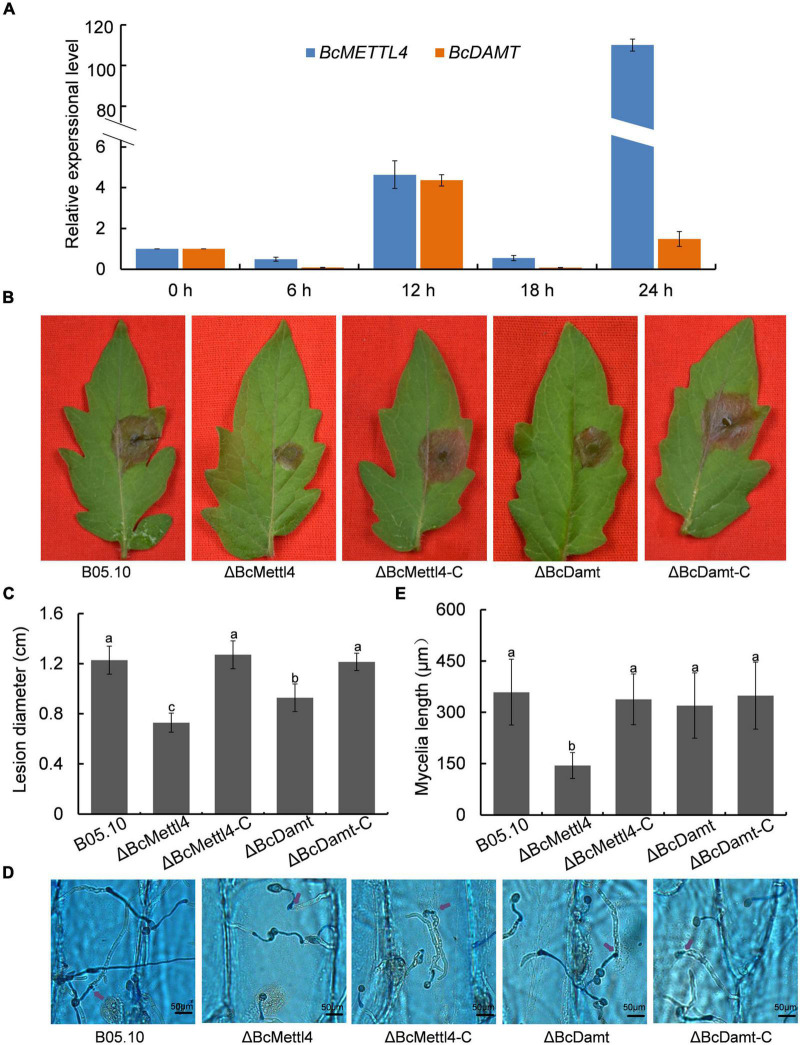
Effects of *BcMETTL4* and *BcDAMT* disruption on virulence and conidia infection. **(A)** The expression levels of *BcMETTL4* and *BcDAMT* at early infection stages evaluated by qRT-PCR. **(B)** Lesions on tomato leaves caused by different strains 72 h after inoculation. **(C)** Lesion diameter of the strains inoculated on tomato leaves. Three experiments were carried out and nine leaves were measured in one biological experiment. Statistical tests were carried out using Tukey’s test. The same letters marked on the bars indicate no significant difference in values at *p* < 0.05. **(D)** Infection of conidia from different strains after 12 h of incubation on onion epidermis. Mycelia growing outside onion cells were colored blue, while mycelia growing inside onion cells are not stained. Penetrated positions are marked by purple arrows. **(E)** Mycelia length of different strains at 12 h after conidia infection on onion epidermis. Data are given as the mean ± SD, *n* = 10. The different letters showed on the columns were significantly different at *p* < 0.05.

The transcript levels of *BcMETTL4* and *BcDAMT* at the point of germination in culture in the presence of tomato leaf tissue to 24 h later were assessed as a proxy for the early interaction stage between *B. cinerea* and tomato leaves. As shown in Figure 3A, the expression levels of *BcMETTL4* and *BcDAMT* remained stable until 12 h and then increased approximately four-fold compared to those at 0 h. However, after 24 h of growth, the expression of *BcMETTL4* increased approximately 110-fold, compared with a twofold increase in *BcDAMT* gene expression (Figure 3A). Highly induced expression of *BcMETTL4* by presence of tomato leaf tissue indicated that BcMettl4 might be involved in the virulence of *B. cinerea*.

The impacts of *BcMETTL4* and *BcDAMT* disruption on the virulence of *B. cinerea* were therefore investigated. First, the mutants ΔBcMettl4 and ΔBcDamt were complemented with the genes *BcMETTL4* and *BcDAMT*, respectively. These two genes including their native promoters and terminators were amplified from wild-type strain B05.10 genomic DNA using the primers listed in Supplementary Table 1. The obtained fragments were inserted into the plasmid pBS-neo and subsequently transformed into the mutants according to the method described above. Finally, two complemented strains, ΔBcMettl4-C and ΔBcDamt-C, were obtained. Deletion of any of the genes, that is, *BcMETTL4* or *BcDAMT*, reduced the pathogenicity of *B. cinerea* on tomato, especially deletion of *BcMETTL4* (Figure 3B). As shown in Figure 3C, the spreading of the lesion induced by the disruption mutant ΔBcMettl4 was significantly reduced compared with that induced by the wild-type strain B05.10 or the disruption mutant ΔBcDamt.

We further examined the ability of these strains to penetrate onion epidermal cells. As shown in Figure 3D, deletion of *BcMETTL4* weakened the infection of mycelia in onion epidermal cells. Mycelia length of different strains of *B. cinerea* at 12 h after conidia infection on onion epidermis were further investigated. It was found that the mycelia length of ΔBcMettl4 invaded onion cells were 144 μm, while those of other strains, B05.10, ΔBcMettl4-C, ΔBcDamt, and ΔBcDamt-C, were 359, 338, 320, and 349 μm (Figure 3E), respectively, revealing that *BcMETTL4* interruption decreased virulence of *B. cinerea* on onion by reducing the conidia infectivity.

### Effects of *BcMETTL4* and *BcDAMT* Disruption on Mycelial Growth, Conidial, Sclerotial Formation and Stress Tolerance

**FIGURE 4:**
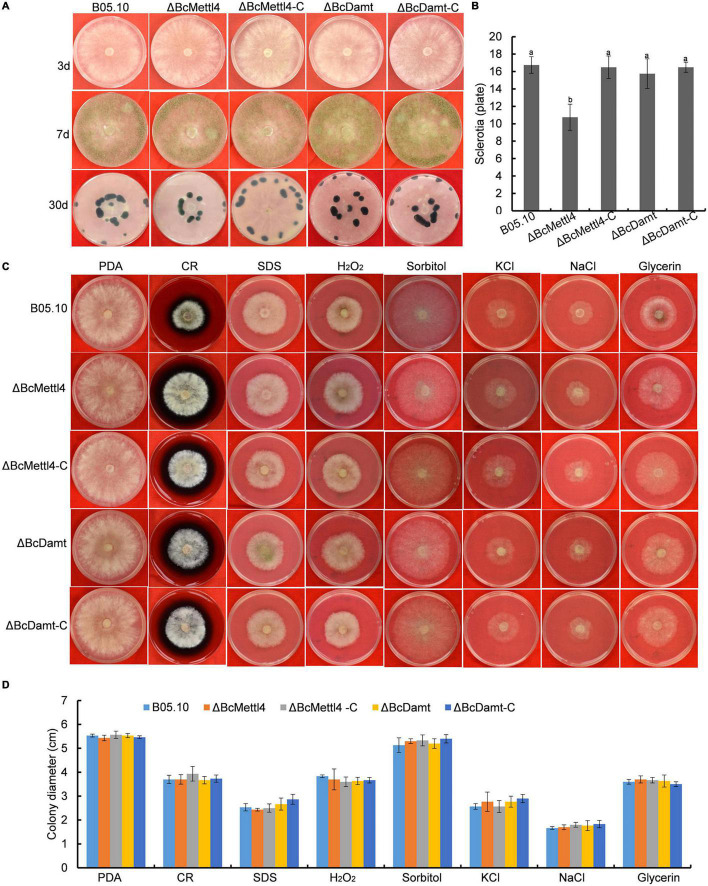
Mycelial growth, conidial and sclerotial formation and stress tolerance of the indicated strains. **(A)** Mycelial growth, conidial and sclerotial development of various strains grown on PDA plates. **(B)** Number of sclerotia produced by various strains grown on PDA plates for 30 days in darkness. Bars denote standard errors from three plates. Tukey’s test was employed to calculate significance. The same letters indicated on the bars mean no significant difference in values at *p* < 0.05. **(C)** The sensitivity of various strains on stress tolerance. **(D)** Colony diameter of the indicated strains grown on various stress conditions. Bars represent standard errors from three plates.

The reduction in genome-wide 6mA (Figure 2D) in the disruption mutant ΔBcMettl4 led to investigations of the biological phenotypes of the different mutations affecting 6mA. After 3 days of cultivation on PDA plates, there were no obvious differences in mycelial growth of B05.10, ΔBcMettl4, ΔBcDamt, ΔBcMettl4-C, and ΔBcDamt-C (Figure 4A). Disruption of *BcMETTL4* or *BcDAMT* also did not affect conidial formation (Figure 4A). However, after growth on PDA plates for 30 days in darkness, ΔBcMettl4 exhibited significantly decreased sclerotial formation compared with B05.10, ΔBcMettl4-C, ΔBcDamt, and ΔBcDamt-C (Figure 4B). Our previous studies have shown that the reduction of sclerotia in *B. cinerea* is often accompanied by a decrease in pathogenicity (Yang et al., 2015; Wang et al., 2020). These observations suggested that the reduction in sclerotia in the *BcMETTL4* mutant might also be correlated with a decrease in the pathogenicity of *B. cinerea*.

The sensitivity of different strains to stress tolerance was also measured. Disruption of *BcMETTL4* or *BcDAMT* did not cause significant sensitivity to cell wall stress, osmotic stabilization, or oxidative stress (Figures 4C,D), indicating that *BcMETTL4* and *BcDAMT* were not involved in the regulation of cell stress tolerance.

### The Point Mutant BcMettl4-APPA Exhibited Phenotypes Similar to Those of ΔBcMettl4

**FIGURE 5:**
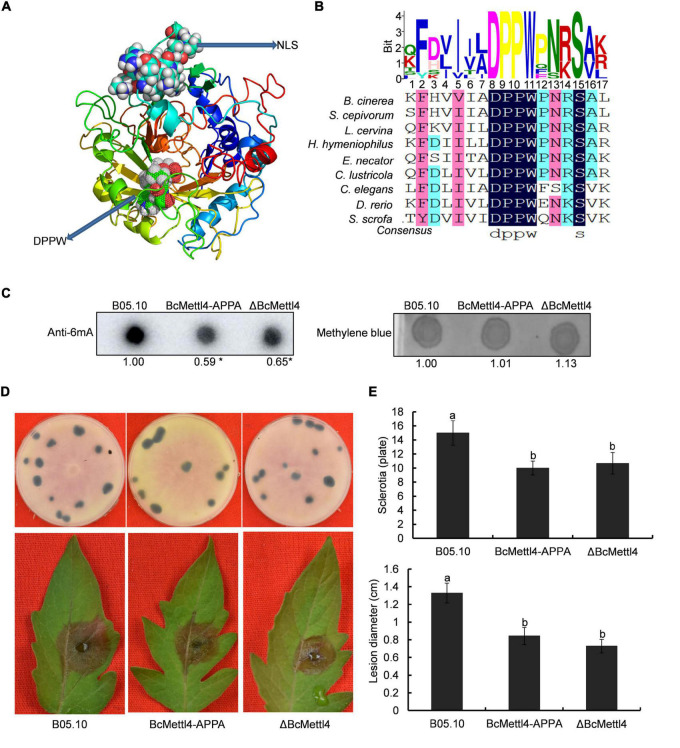
Impact of BcMettl4 loss-of-function on DNA modification, pathogenicity, and sclerotia formation of *B. cinerea*. **(A)** Three-dimensional structure of BcMettl4 modeled were predicted by I-TASSER. **(B)** Sequence alignment of motifs in methyltransferase form *B. cinerea*, *Sclerotium cepivorum*, *Lachnellula cervine*, *Hyphodiscus hymeniophilus*, *Erysiphe necator*, *Coniella lustricola*, *Caenorhabditis elegans*, *Danio rerio*, and *Sus scrofa*. **(C)** Dot blot assay of 6mA in the indicated strains. A total of 200 ng DNA was investigated on each dot. The relative ratio of 6mA in wild-type strain B05.10 and point mutant BcMettl4-APPA was calculated by ImageJ. The asterisk represents significant difference at *p* < 0.05. **(D)** The disease lesions caused by various strains following inoculation on tomato leaves for 3 days and the sclerotia formation of various strains after grown in darkness for 30 days. **(E)** Lesion diameter of the strains inoculated on tomato leaves and the number of sclerotia in various stains. Error-bars were from three experiments. For lesion diameter, nine leaves were investigated in one biological experiment. Three plates were used to count sclerotia. Tukey’s test was used to calculate significance. The same letters marked on the columns were not significantly different at *p* < 0.05.

Methyltransferases contain a conserved catalytic motif, “DPPW.” A 3-dimensional structure of BcMettl4 was constructed, and the signature motif “DPPW” was found to be located in the catalytic domain (Figure 5A). Multiple sequence alignments of the catalytic motifs in 6mA methyltransferases were performed. The results showed that the conserved catalytic motif “DPPW” in BcMettl4 exhibited high similarity with Mettl4 orthologs (Figure 5B). To study the catalytic function of BcMettl4, the catalytic domain “DPPW” was mutated to “APPA” in BcMettl4. After the mutation to “APPA,” the methylation level of DNA in *B. cinerea* decreased (Figure 5C) and both virulence and sclerotium formation decreased (Figures 5D,E), which was consistent with the phenotype of the ΔBcMettl4 mutant. These data suggested that the catalytic motif “DPPW” plays a key role in the enzymatic function of BcMettl4 and that BcMettl4 is involved in the regulation of DNA methylation levels and virulence.

### Genomic Profiling of 6mA in *Botrytis cinerea*

**FIGURE 6:**
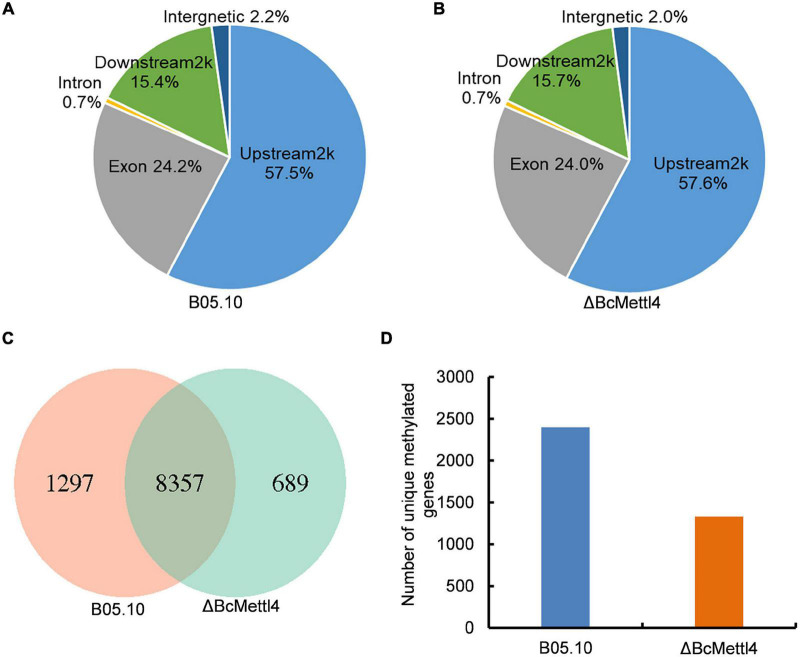
The 6mA methylated genes in *B. cinerea*. **(A)** Peak distribution in genomic elements of the wild-type strain B05.10. **(B)** Peak distribution in genomic elements of the disruption mutant ΔBcMettl4. **(C)** Venn diagram comparing unique 6mA peaks between the wild-type B05.10 and the mutant ΔBcMettl4. **(D)** The number of unique methylated genes in wild-type strain B05.10 and the disruption mutant ΔBcMettl4.

To detect naturally occurring methylated genes globally regulated by *BcMETTL4* in *B. cinerea*, we performed a MeDIP-Seq analysis of the 6mA methylomes of the disruption mutant ΔBcMettl4 and its wild-type strain B05.10. The expression of *BcMETTL4* increased significantly after 24 h infection (Figure 3A). We therefore collected the samples of conidial germination at 24 h for MeDIP-seq. Three biological replicates for each strain of ΔBcMettl4 and B05.10 were investigated in MeDIP-Seq experiments. A total of 30,320 6mA-enriched regions (peaks) were captured from the three B05.10 biological replicates, while a total of 28,385 methylation peaks were obtained from the three ΔBcMettl4 biological replicates (Supplementary Table 2). These observations further confirmed that disruption of *BcMETTL4* reduced the methylation level of *B. cinerea*. After assembling the attained sequences, it was found that most of the 6mA peaks (57%) were distributed in the upstream regions of genes, followed by 24% in the exon, and 15% in the downstream regions in wild-type B05.10 and the mutant ΔBcMettl4 (Figures 6A,B). The 6mA peaks were further investigated and wild-type B05.10 exhibited 1,297 unique 6mA peaks while 689 unique 6mA peaks were identified in the mutant ΔBcMettl4 (Figure 6C). There were 8,357 6mA peaks that were found in both the wild-type B05.10 and the mutant ΔBcMettl4 (Figure 6C). Among the unique 6mA peaks of wild-type B05.10, 2,396 genes were detected (Figure 6D and Supplementary Table 3). However, only 1,330 genes were detected in the unique 6mA peaks of the mutant ΔBcMettl4 (Figure 6D and Supplementary Table 4).

### *BcMETTL4* Inactivation Downregulated the Expression of 13 Methylated Genes in Which Methylation Occurred in the Promoter Region

Since the majority of the 6mA peaks were identified in the regions upstream of the affected genes (Figures 6A,B), involvement of the promoter regions and regulation of gene expression was indicated. To identify all genes that might exhibit differential expression due to the disruption of *BcMETTL4* in *B. cinerea*, we performed an RNA-Seq analysis of the full transcriptomic profiles of the wild-type strain B05.10 and the disruption mutantΔBcMettl4. The differentially expressed genes were further analyzed using DESeq software (Wang et al., 2010). A total of 792 genes, including 601 upregulated genes (Supplementary Table 5) and 191 downregulated genes (Supplementary Table 6), were found to be differentially expressed in the *BcMETTL4* mutant compared with the wild-type strain B05.10, which accounted for 4.82% (792/16,448) of the annotated genes in *B. cinerea*.

**FIGURE 7:**
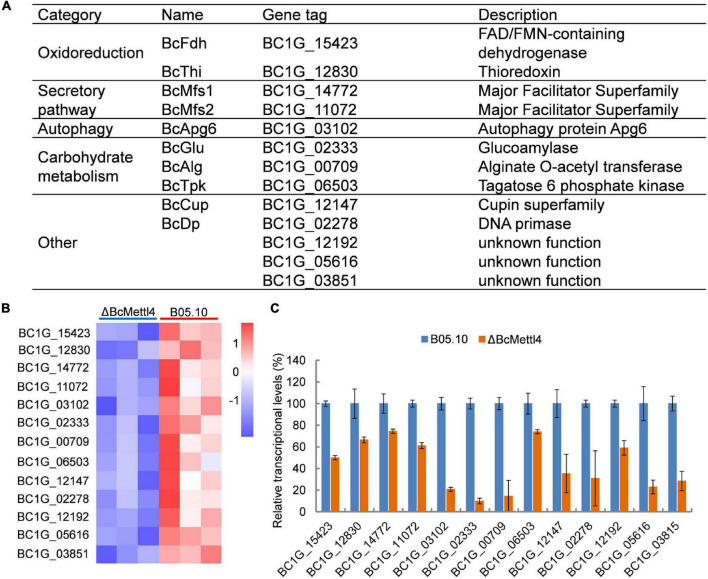
The downregulated methylated genes in which modification occurs in the promoter region. **(A)** Categories of the screened genes. **(B)** RNA-Seq analysis of 13 methylated genes. The colored squares show the level of gene expression referred to log2 FPKM value in each of the three replicas. **(C)** Validation of RNA-Seq data of selected genes by fluorescent real-time qRT-PCR. The reference gene used in this study was Beta-tubulin. Data are given as the mean ± SD, *n* = 3.

To test the hypothesis that the unique genes modified at promoter sites might be directly regulated by BcMettl4, the transcription levels of the unique genes were further analyzed in the mutant ΔBcMettl4. After disruption of *BcMETTL4*, among the unique methylated genes modified at promoter sites in the wild-type strain B05.10, 32 genes were upregulated (Supplementary Table 7) and 13 genes were downregulated (Figures 7A,B). Since the virulence of the *BcMETTL4* mutant was significantly reduced, we hypothesized that the 13 methylated genes modified in the promoter region with downregulated expression might be associated with pathogenicity. The genes shown in Figure 7A were therefore subsequently further analyzed.

The reduced transcript levels of the 13 genes shown in Figure 7B were further validated by fluorescent real-time qRT-PCR (Figure 7C). As shown in Figure 7A, these methylated genes were divided into five categories, including oxidoreduction, secretory pathways, autophagy and carbohydrate metabolism, according to their biological functions. It has been well documented that proteins related to oxidative stress sensitivity (Yang et al., 2015), autophagy (Ren et al., 2018), and secretion (Zhang et al., 2016) are important factors affecting the virulence of *B. cinerea*. These observations indicated that the downregulated methylated genes related to oxidoreduction, secretory pathways and autophagy may cause a decrease in the pathogenicity of *B. cinerea*.

### The Roles of BcMfs2 and BcFdh in the Virulence of *Botrytis cinerea*

**FIGURE 8:**
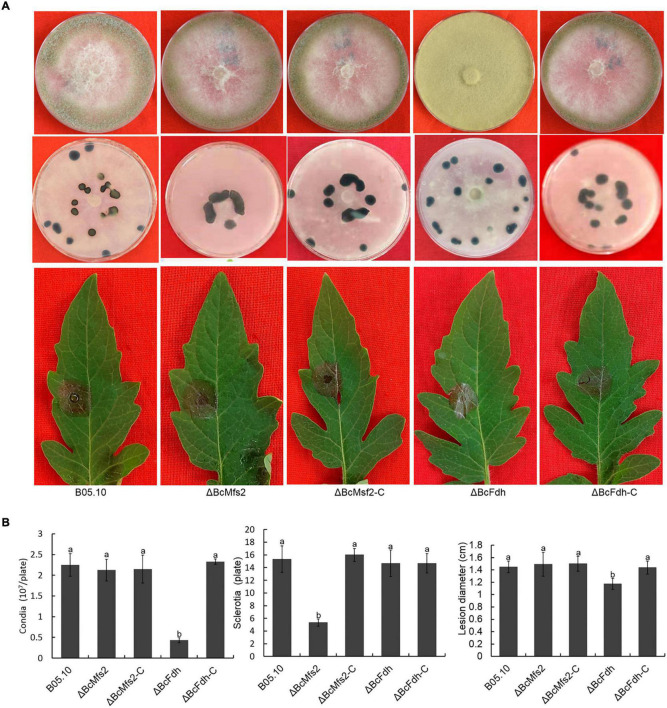
Impact of *BcMFS2* and *BcFDH* deletion on pathogenicity of *B. cinerea*. **(A)** Effect of deletions *BcMFS2* or *BcFDH* on conidia biosynthesis, sclerotia formation and virulence. **(B)** Quantification of conidia, sclerotia and pathogenicity of different strains. Bars represent standard errors from three experiments. In each biological experiment, lesion diameters of nine leaves were investigated. Three plates were used to count sclerotia and conidia. Statistical tests were carried out using Tukey’s test. The different letters marked on the columns indicate statistical significance *p* < 0.05.

Deletion of *BcMETTL4* both reduced the methylation and expression levels of 13 genes (Figure 7). We speculated that these genes might be involved in virulence of *B. cinerea*. Two of them were randomly selected for targeted disruption. *BcMFS2* encoding a major facilitator superfamily (MFS) transporter and *BcFDH* encoding a FAD/FMN-containing dehydrogenase were independently deleted in the wild-type strain B05.10. Two mutants, ΔBcMfs2 and ΔBcFdh, were subsequently obtained. The genes *BcMFS2* and *BcFDH* were also cloned and complemented both mutants, resulting in transformants, ΔBcMfs2-C and ΔBcFdh-C, respectively. Deletion of *BcMFS2* reduced sclerotium formation (Figures 8A,B). Further infection assays of tomato leaves were performed, and the results showed that deletion of *BcMFS2* did not reduce the virulence of *B. cinerea* (Figures 8A,B). These findings indicated that *BcMFS2* was involved in sclerotium formation but not in the virulence of *B. cinerea*. Another interesting finding was that deletion of *BcFDH* severely hindered conidium formation in *B. cinerea* (Figure 8B). Infection of tomato leaves by ΔBcFdh was further performed, and the results showed that deletion of the *BcFDH* gene reduced the pathogenicity of *B. cinerea* by 20% (Figure 8B). These observations demonstrated that *BcFDH* was involved in both conidium formation and the pathogenicity of *B. cinerea*.

## Discussion

As a basic epigenetic marker, DNA methylation has been widely documented in both eukaryotes and prokaryotes (Bird, 2007; Vasu and Nagaraja, 2013). However, its characteristics in *B. cinerea* are still elusive. In this study, we found that 6mA is a widespread DNA modification in the *B. cinerea* genome. Previous studies have shown that DNA methylation levels are dynamic and controlled by two enzymes, methyltransferase and demethylase (Greer et al., 2015; Zhang et al., 2015). MT-A70 is an important 6mA methyltransferase (Bujnicki et al., 2002). Gray mold contains two homologous methyltransferases, BcMettl4 (Figure 1A) and BcDamt (Figure 1B). As shown in Figure 2D, disruption of *BcMETTL4* but not *BcDAMT* resulted in a decrease in the DNA methylation level of *B. cinerea*, suggesting that BcMettl4 is related to 6mA modification. Previous reports have shown that the signature motif “DNSH/PP/YFW” of 6mA methyltransferase is responsible for substrate binding and catalytic activity (Iyer et al., 2016; Wang et al., 2019). After the mutation of “DPPW” to “APPA,” the methylation level in *B. cinerea* decreased (Figure 5C), demonstrating a catalytic role of “DPPW” in BcMettl4. Consistent with these results, the *AMT1* APPA mutant of *Tetrahymena thermophila* had a reduced 6mA level (Wang et al., 2019). Unlike the genome of *B. cinerea*, the genome of *Phytophthora sojae* contains three 6mA methyltransferase genes (*DAMT1*, *DAMT2*, and *DAMT3*), and single knockouts of any of these genes in the *P. sojae* strain P6497 resulted in a significant decrease in DNA methylation (Chen et al., 2018).

Using MeDIP-Seq analysis, we identified a large number of 6mA peaks that were distributed in the genome of *B. cinerea*. As shown in Figures 6A,B, 6mA was significantly enriched in upstream and downstream gene regions in both the wild-type strain B05.10 and the disruption mutant ΔBcMettl4. Consistent with our observations, almost all of the 6mA were found in the promoter regions, either at or slightly downstream of the transcription start sites in early-diverging fungi, *Hesseltinella vesiculosa*, *Absidia repens*, *Lobosporangium transversale*, and *Syncephalastrum racemosum* (Mondo et al., 2017). Several other studies also revealed that the transcription initiation sites of *Chlamydomonas* (Fu et al., 2015) and *Phytophthora* (Chen et al., 2018) were readily modified by 6mA. In *Arabidopsis*, the 6mA modification level in gene bodies was higher than that of intergenic regions (Liang et al., 2018). Similar to the result for *Arabidopsis*, DNA 6mA modification was preferentially distributed in the gene bodies rather than the intergenomic regions in *Tetrahymena* (Wang et al., 2017). However, 6mA was evenly distributed on chromosomes and correlated with 5mC distribution in rice (Zhou et al., 2018). Inconsistent with the above findings, DNA 6mA modification was significantly enriched in exon regions in the human genome (Xiao et al., 2018). Taken together, the results suggested that the genomic localization pattern of DNA 6mA modification in different species varies greatly, indicating that these modifications are hitherto unknown epigenetic markers.

*Botrytis cinerea* is an important necrotizing pathogen. It has been well documented that cellular accumulation of reactive oxygen species (ROS) is one of the immune responses of plants after infection by pathogens (Kaku et al., 2006; Cheval et al., 2013; Wada et al., 2019). Previous studies have documented that the virulence of pathogens is often related to resistance to oxidative stress (Yan et al., 2011; Yang et al., 2015, 2018). In a previous study, we found that knocking out the catalase *BcCAT* (*BC1G_12146*) reduced the toxicity of gray mold (Wang et al., 2020). In this study, *BcCAT* was downregulated in the disruption mutant ΔBcMettl4 (Supplementary Table 6). We also found that three oxidative stress-related genes, *BcCATA* (*BC1G_01095*), *BcCAT3* (*BC1G_02407*), and *BcCAT4* (*BC1G_09386*), were upregulated in ΔBcMettl4 (Supplementary Table 5), which might compensate for the effect of downregulation of BcCAT. After *BcMETTL4* was inactivated, two methylated genes, *BcFDH* and *BcTHI*, which are related to oxidoreduction, were also downregulated (Figure 7A). Further experiments confirmed that knockout of *BcFDH* reduced the pathogenicity of *B. cinerea* (Figure 8B). The disruption of *BcFDH* severely hindered conidia formation (Figure 8B). The genome of *B. cinerea* contains 16 genes involved in conidiation (Amselem et al., 2011). We found that only one of them, *BcPPOA80* (*BC1G_14780*), were downregulated when *BcMETTL4* was deleted (Supplementary Table 6), indicating that the downregulation of only *BcPPOA80* might have little effect on conidia formation. However, it is still completely unknown why the conidia formation was severely hindered when the *BcFDH* gene was deleted.

Two methylated genes, *BcMFS1* and *BcMFS2*, which are associated with MFS transporters, were downregulated in the mutant ΔBcMettl4 (Figure 7A). After *BcMFS2* was deleted, the mutant’s ability to form sclerotia was reduced (Figure 8A). BcSec14 and BcSec31 are involved in vesicle transport, and deletion of BcSec14 and BcSec31 resulted in a reduction in pathogenicity and protein secretion in gray mold (Zhang et al., 2016). It has been well documented that MFS is the largest group of membrane transporters (Law et al., 2008). MFS transporters are also involved in cell secretion and can transport some secondary metabolites and compounds (Vela-Corcía et al., 2019). The polysaccharide components of the cell wall are mainly hydrolyzed into monosaccharides and oligosaccharides by degrading enzymes, thereby reducing the protection of the cell wall against pathogens and increasing the ability of pathogens to infect plants (Cantarel et al., 2009; Kubicek et al., 2014). A cell polysaccharide-degrading enzyme-encoding gene, *BcGLU*, was found among the downregulated methylated genes (Figure 7A); this gene may be involved in the pathogenicity of *B. cinerea*. Taken together, these findings suggest that BcMettl4 may regulate pathogenicity via its effect on multiple pathways.

## Conclusion

DNA adenine N^6^-methylation sites are widely distributed in the genome of *B. cinerea*. BcMettl4 is nuclear-localized and contains the conserved motif “DPPW,” which is likely related to its interaction with DNA for methylation modification. MeDIP-Seq showed that the regions 2,000 bp upstream and downstream of the genes were preferentially modified by methylation in *B. cinerea*. *BcMETTL4* disruption and mutation of “DPPW” to “APPA” both resulted in a significant reduction in the genomic methylation and pathogenicity of *B. cinerea*. These observations indicate that 6mA provides potential epigenetic markers in *B. cinerea* and that BcMettl4 regulates the level of DNA methylation in this important plant pathogen. Our results extend the current understanding of the global regulatory role of 6mA in filamentous plant pathogens.

## Data Availability Statement

The data has been made available at NCBI with accession numbers PRJNA817858 and PRJNA817835.

## Author Contributions

ZM, GW, and WL contributed to the conception and design of the study. ZM, GW, and XW performed the experiments. ZM, GW, HS, and XW organized the database. ZM, HS, and XW performed the statistical analysis. ZM, GW, and WL wrote the first draft of the manuscript. GW, WL, and DG reviewed and edited the manuscripts. All authors provided comments on the manuscript and approved the submitted version.

## Conflict of Interest

The authors declare that the research was conducted in the absence of any commercial or financial relationships that could be construed as a potential conflict of interest.

## Publisher’s Note

All claims expressed in this article are solely those of the authors and do not necessarily represent those of their affiliated organizations, or those of the publisher, the editors and the reviewers. Any product that may be evaluated in this article, or claim that may be made by its manufacturer, is not guaranteed or endorsed by the publisher.

## Supplementary Material

Supplementary Figure 1 Disruption of *BcMETTL4* and validation of three different mutants by PCR. **(A)** Marker exchange technique used for disruption of *BcMETTL4*. **(B)** PCR products with Mettl4-in-F/R (the first four lanes except Marker) or Mettl4-out-F/R (the last four lanes) from the genomic DNAs of wild-type (lanes 1 and 5) and ΔBcMettl4 (three different mutants).

Supplementary Figure 2 Work procedure for *BcDAMT* deletion. **(A)** Marker exchange technique used for disruption of *BcDAMT*. **(B)** PCR products with DAMT-in-F/R (the first four lanes except Marker) or DAMT-out-F/R (the last four lanes) from the genomic DNAs of wild-type (lanes 1 and 5) and ΔBcDamt (three different mutants).
