# Supplementary figures and images for: BcMettl4-Mediated DNA Adenine N6-Methylation Is Critical for Virulence of Botrytis cinerea
Source: Front Microbiol. 2022 Jun 30;13:925868. doi: 10.3389/fmicb.2022.925868 (PMC9279130; doi:10.3389/fmicb.2022.925868)

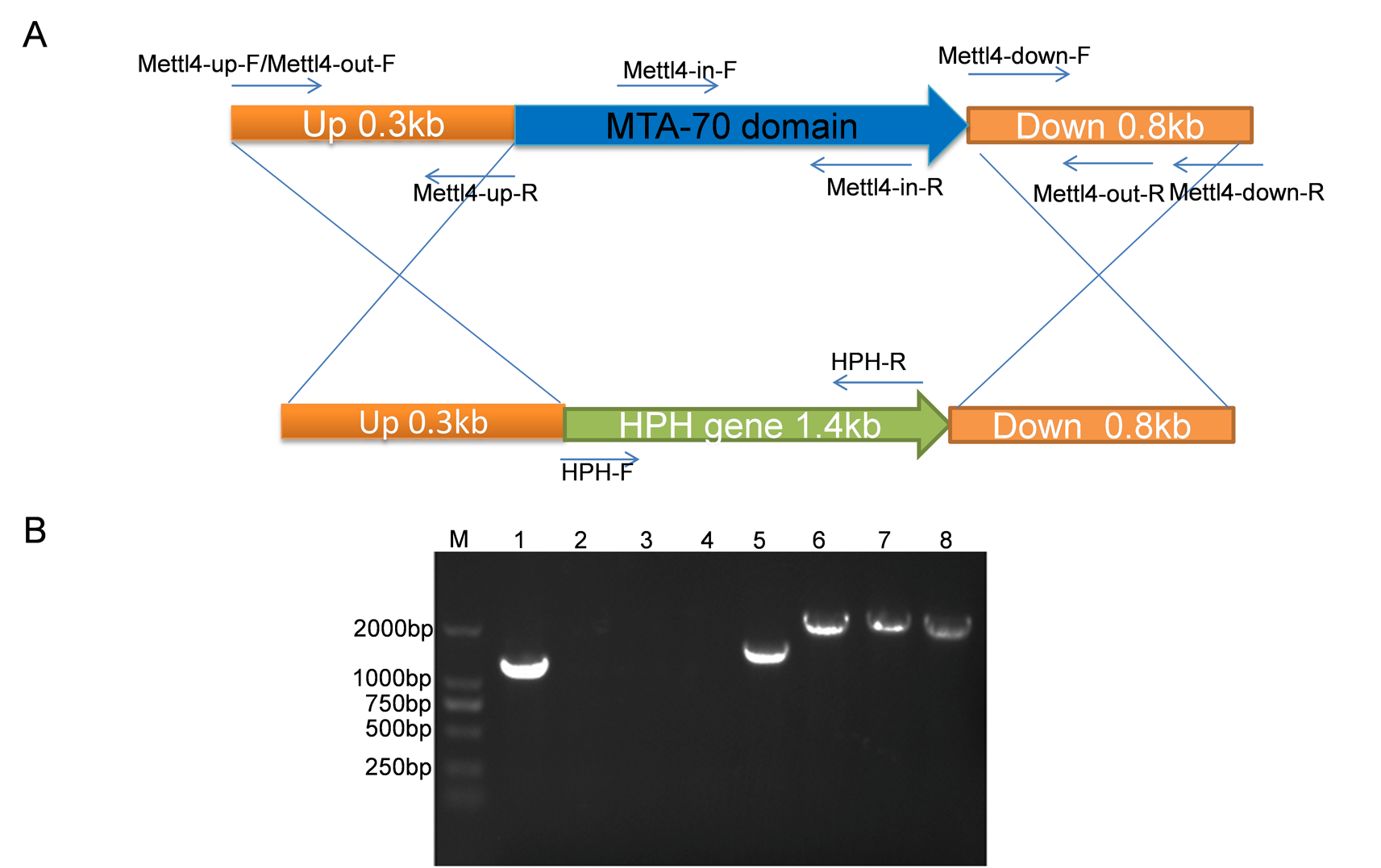

Supplement: Supplementary Figure 1 — Disruption of BcMETTL4 and validation of three different mutants by PCR. (A) Marker exchange technique used for disruption of BcMETTL4. (B) PCR products with Mettl4-in-F/R (the first four lanes except Marker) or Mettl4-out-F/R (the last four lanes) from the genomic DNAs of wild-type (lanes 1 and 5) and ΔBcMettl4 (three different mutants). [file Image_1.TIF]

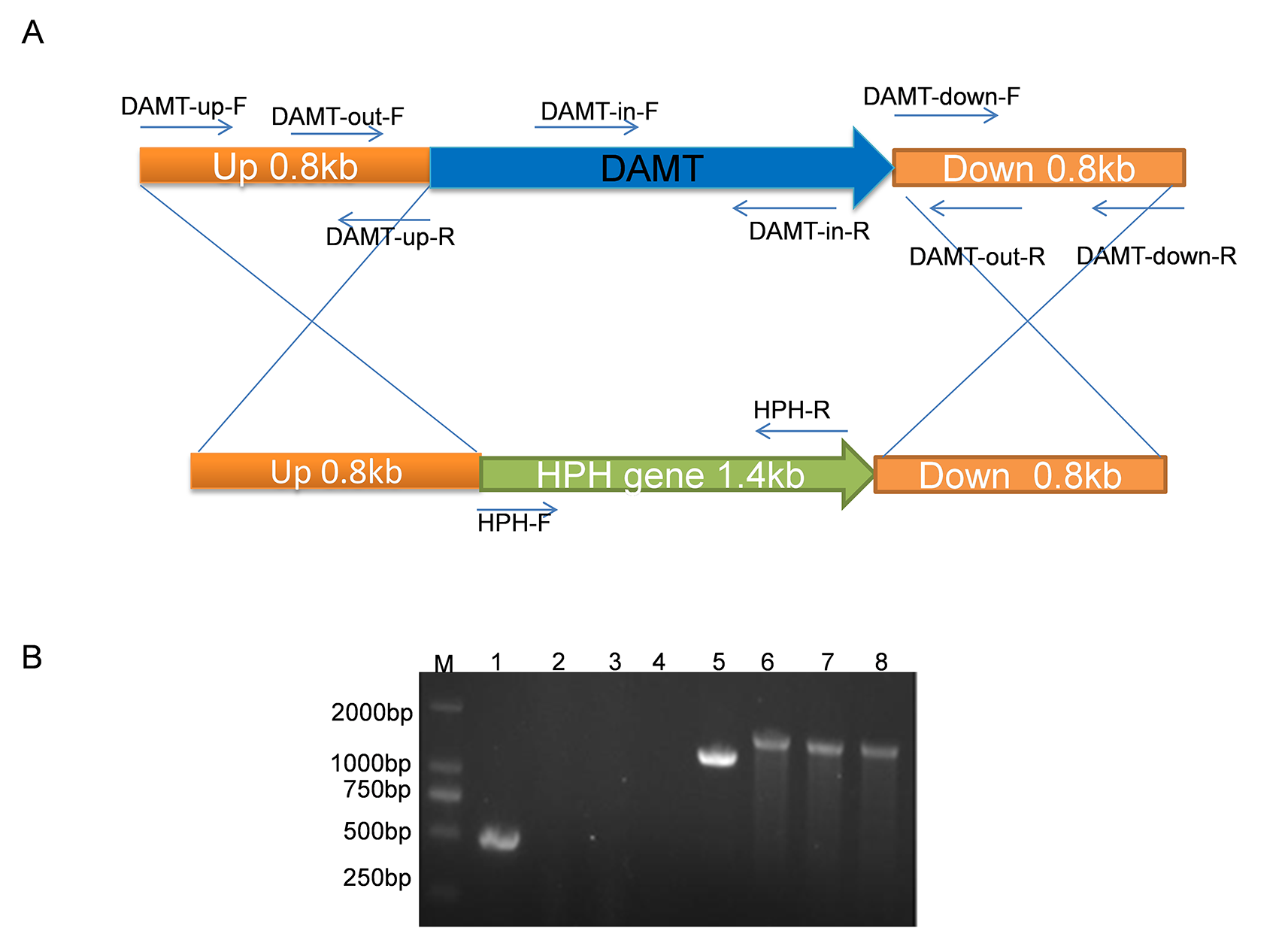

Supplement: Supplementary Figure 2 — Work procedure for BcDAMT deletion. (A) Marker exchange technique used for disruption of BcDAMT. (B) PCR products with DAMT-in-F/R (the first four lanes except Marker) or DAMT-out-F/R (the last four lanes) from the genomic DNAs of wild-type (lanes 1 and 5) and ΔBcDamt (three different mutants). [file Image_2.TIF]
